# Supplementary material for: Neuromelanin accumulation drives endogenous synucleinopathy in non-human primates
Source: Brain. 2023 Sep 28;146(12):5000–14. doi: 10.1093/brain/awad331 (PMC10689915; doi:10.1093/brain/awad331)
Supplement: awad331_Supplementary_Data [file awad331_supplementary_data.zip › awad331 Supplementary Table 2.pdf]

Supplementary Table 2: List of reagents

ITEM	DILUTION	INCUBATION TIME	SOURCE	IDENTIFIER	RRID Citation
**Antibodies**					
Goat anti-Tyrosine Hydrox. (polyclonal)	1:500	Overnight	My Biosource	Cat# MBS421729	
Mouse anti-α-synuclein (monoclonal)	1:40	Overnight	Leica	Cat# NCL-L-ASYN	AB_442103
Mouse anti-P-α-syn (monoclonal)	1:1000	Overnight	Fujifilm Wako	Cat# 015-25191	AB_2537218
Guinea Pig anti-P62 (polyclonal)	1:1000	Overnight	PROGEN	Cat# GP62-C	AB_2687531
Mouse anti-Ubiquitin (monoclonal)	1:500	Overnight	Abcam	Cat# ab7254	AB_305802
Rabbit anti-NeuN (monoclonal)	1:1000	Overnight	Abcam	Cat# ab177487	AB_2532109
Rabbit anti-Iba 1 (polyclonal)	1:500	Overnight	Fujifilm Wako	Cat# 019-19741	AB_839504
Mouse anti-CD68 (monoclonal)	1:300	Overnight	Dako	Cat# M0814	AB_2314148
Donkey anti-Goat (Biotin-SP)	1:600	120 min	Jackson	Cat# 705-065-147	AB_2340397
Donkey anti-Goat (Alexa488)	1:200	120 min	Invitrogen	Cat# A-11055	AB_2534102
Donkey anti-Goat (Alexa350)	1:200	120 min	Invitrogen	Cat# A-21081	AB_2535738
Donkey anti-Goat (Alexa633)	1:200	120 min	Invitrogen	Cat# A-21082	AB_2535739
Donkey anti-Guinea Pig (Alexa594)	1:200	120 min	Jackson	Cat# 706-585-148	AB_2340474
Donkey anti-Mouse (Alexa488)	1:200	120 min	Invitrogen	Cat# A-21202	AB_141607
Donkey anti-Mouse (Alexa546)	1:200	120 min	Invitrogen	Cat# A-10036	AB_2534012
Donkey anti-Rabbit (Alexa488)	1:200	120 min	Invitrogen	Cat# A-21206	AB_2535792
**Commercial assays**					
ABC kit standard		60 min	Vector Labs	Cat# PK4000	
Neutral Red	0.40%	1 min	Sigma	Cat# 72210	
V-VIP		1 min	Vector Labs	Cat# SK-4600	
Proteinase K	1 µg/ml	10 min	Invitrogen	Cat# 25530049	
DAPI	1:50000	5 min	Invitrogen	Cat# D1306	
