# Supplementary material for: Neuromelanin accumulation drives endogenous synucleinopathy in non-human primates
Source: Brain. 2023 Sep 28;146(12):5000–14. doi: 10.1093/brain/awad331 (PMC10689915; doi:10.1093/brain/awad331)
Supplement: awad331_Supplementary_Data [file awad331_supplementary_data.zip › brain-2023-00752-File010.pdf]

Supplementary Figure 2. Sequence for pAAV-CMV-hTyr

pAAV-CMV-Tyr Sequence

CAGCAGCTGCGCGCTCGCTCGCTCACTGAGGCCGCCCGGGCAAAGCCCGGGCGTCGGGC
GACCTTTGGTCGCCCGGCCTC
AGTGAGCGAGCGAGCGCGCAGAGAGGGAGTGGCCAACTCCATCACTAGGGGTTCCTTGT
AGTTAATGATTAACCCGCCAT
GCTACTTATCTACGTAGCCATGCTCTAGACATGGCTCGACAGATCTCAATATTGGCCAT
TAGCCATATTATTCATTGGTT
ATATAGCATAAATCAATATTGGCTATTGGCCATTGCATACGTTGTATCTATATCATAAT
ATGTACATTTATATTGGCTCA
TGTCCAATATGACCGCCATGTTGGCATTGATTATTGACTAGTTATTAATAGTAATCAAT
TACGGGGTCATTAGTTCATAG
CCCATATATGGAGTTCCGCGTTACATAACTTACGGTAAATGGCCCGCCTGGCTGACCGC
CCAACGACCCCCGCCCATTGA
CGTCAATAATGACGTATGTTCCCATAGTAACGCCAATAGGGACTTTCCATTGACGTCAA
TGGGTGGAGTATTTACGGTAA
ACTGCCCACTTGGCAGTACATCAAGTGTATCATATGCCAAGTCCGCCCCCTATTGACGT
CAATGACGGTAAATGGCCCGC
CTGGCATTATGCCCAGTACATGACCTTACGGGACTTTCCTACTTGGCAGTACATCTACG
TATTAGTCATCGCTATTACCA
TGGTGATGCGGTTTTGGCAGTACACCAATGGGCGTGGATAGCGGTTTGACTCACGGGGA
TTTCCAAGTCTCCACCCCATT
GACGTCAATGGGAGTTTGTTTTGGCACCAAAATCAACGGGACTTTCCAAAATGTCGTAA
CAACTGCGATCGCCCGCCCCG
TTGACGCAAATGGGCGGTAGGCGTGTACGGTGGGAGGTCTATATAAGCAGAGCTCGTTT
AGTGAACCGTCAGATCACTAG
AAGCTTTATTGCGGTAGTTTATCACAGTTAAATTGCTAACGCAGTCAGTGCTTCTGACA
CAACAGTCTCGAACTTAAGCT
GCAGTGACTCTCTTAAGGTAGCCTTGCAGAAGTTGGTCGTGAGGCACTGGGCAGGTAAG
TATCAAGGTTACAAGACAGGT
TTAAGGAGACCAATAGAAACTGGGCTTGTCGAGACAGAGAAGACTCTTGCGTTTCTGAT
AGGCACCTATTGGTCTTACTG
ACATCCACTTTGCCTTTCTCTCCACAGGTGTCCACTCCCAGTTCAATTACAGCTCTTAA
GGCTAGAGTACTTAATACGAC
TCACTATAGGCTAGCctcgacctcgagacgcgtgataaacttaagcttggtaccgagct
cggatccactagtccagtgtg
gtggaattctgcagatatccagcacagtggcggccgctcgaccGACCTTGTGAGGACTA
GAGGAAGAATGCTCCTGGCTG
TTTTGTACTGCCTGCTGTGGAGTTTCCAGACCTCCGCTGGCCATTTCCCTAGAGCCTGT
GTCTCCTCTAAGAACCTGATG
GAGAAGGAATGCTGTCCACCGTGGAGCGGGGACAGGAGTCCCTGTGGCCAGCTTTCAGG
CAGAGGTTCCTGTCAGAATAT
CCTTCTGTCCAATGCACCACTTGGGCCTCAATTTCCCTTCACAGGGGTGGATGACCGGG
AGTCGTGGCCTTCCGTCTTTT
ATAATAGGACCTGCCAGTGCTCTGGCAACTTCATGGGATTCAACTGTGGAAACTGCAAG
TTTGGCTTTTGGGGACCAAAC
TGCACAGAGAGACGACTCTTGGTGAGAAGAAACATCTTCGATTTGAGTGCCCCAGAGAA
GGACAAATTTTTTGCCTACCT

CACTTTAGCAAAGCATACCATCAGCTCAGACTATGTCATCCCCATAGGGACCTATGGCC
AAATGAAAAATGGATCAACAC
CCATGTTTAACGACATCAATATTTATGACCTCTTTGTCTGGATGCATTATTATGTGTCA
ATGGATGCACTGCTTGGGGGA
TCTGAAATCTGGAGAGACATTGATTTTGCCCATGAAGCACCAGCTTTTCTGCCTTGGCA
TAGACTCTTCTTGTTGCGGTG
GGAACAAGAAATCCAGAAGCTGACAGGAGATGAAAACTTCACTATTCCATATTGGGACT
GGCGGGATGCAGAAAAGTGTG
ACATTTGCACAGATGAGTACATGGGAGGTCAGCACCCCACAAATCCTAACTTACTCAGC
CCAGCATCATTCTTCTCCTCT
TGGCAGATTGTCTGTAGCCGATTGGAGGAGTACAACAGCCATCAGTCTTTATGCAATGG
AACGCCCGAGGGACCTTTACG
GCGTAATCCTGGAAACCATGACAAATCCAGAACCCCAAGGCTCCCCTCTTCAGCTGATG
TAGAATTTTGCCTGAGTTTGA
CCCAATATGAATCTGGTTCCATGGATAAAGCTGCCAATTTCAGCTTTAGAAATACACTG
GAAGGATTTGCTAGTCCACTT
ACTGGGATAGCGGATGCCTCTCAAAGCAGCATGCACAATGCCTTGCACATCTATATGAA
TGGAACAATGTCCCAGGTACA
GGGATCTGCCAACGATCCTATCTTCCTTCTTCACCATGCATTTGTTGACAGTATTTTTG
AGCAGTGGCTCCGAAGGCACC
GTCCTCTTCAAGAAGTTTATCCAGAAGCCAATGCACCCATTGGACATAACCGGGAATCC
TACATGGTTCCTTTTATACCA
CTGTACAGAAATGGTGATTTCTTTATTTCATCCAAAGATCTGGGCTATGACTATAGCTA
TCTACAAGATTCAGACCCAGA
CTCTTTTCAAGACTACATTAAGTCCTATTTGGAACAAGCGAGTCGGATCTGGTCATGGC
TCCTTGGGGCGGCGATGGTAG
GGGCCGTCCTCACTGCCCTGCTGGCAGGGCTTGTGAGCTTGCTGTGTCGTCACAAGAGA
AAGCAGCTTCCTGAAGAAAAG
CAGCCACTCCTCATGGAGAAAGAGGATTACCACAGCTTGTATCAGAGCCATTTATAAAA
GGCTTAGGCAATAGAGTAGGG
CCAAAAAGCCTGACCTCACTCTAACTCAAAGTAATGTCCAGGTTCCCAGAGAATATCTG
CTGGTATTTTTCTGTAAAGAC
CATTTGCAAAATTGTAACCTAATACAAAGTGTAGCCTTCTTCCAACTCAGGTAGAACAC
ACCTGTCTTTGTCTTGCTGTT
TTCACTCAGCCCTTTTAACATTTTCCCCTAAGCCCctagagggcccgtttatcggatcc
cggccggcggccgcTTCCCTT
TAGTGAGGGTTAATGCTTCGAGCAGACATGATAAGATACATTGATGAGTTTGGACAAAC
CACAACTAGAATGCAGTGAAA
AAAATGCTTTATTTGTGAAATTTGTGATGCTATTGCTTTATTTGTAACCATTATAAGCT
GCAATAAACAAGTTAACAACA
ACAATTGCATTCATTTTATGTTTCAGGTTCAGGGGGAGATGTGGGAGGTTTTTTAAAGC
AAGTAAAACCTCTACAAATGT
GGTAAAATCCGATAAGGGACTAGAGCATGGCTACGTAGATAAGTAGCATGGCGGGTTAA
TCATTAACTACAAGGAACCCC
TAGTGATGGAGTTGGCCACTCCCTCTCTGCGCGCTCGCTCGCTCACTGAGGCCGGGCGA
CCAAAGGTCGCCCGACGCCCG
GGCTTTGCCCGGGCGGCCTCAGTGAGCGAGCGAGCGCGCCAGCTGGCGTAATAGCGAAG
AGGCCCGCACCGATCGCCCTT
CCCAACAGTTGCGCAGCCTGAATGGCGAATGGAATTCCAGACGATTGAGCGTCAAAATG
TAGGTATTTCCATGAGCGTTT

TTCCGTTGCAATGGCTGGCGGTAATATTGTTCTGGATATTACCAGCAAGGCCGATAGTT
TGAGTTCTTCTACTCAGGCAA
GTGATGTTATTACTAATCAAAGAAGTATTGCGACAACGGTTAATTTGCGTGATGGACAG
ACTCTTTTACTCGGTGGCCTC
ACTGATTATAAAAACACTTCTCAGGATTCTGGCGTACCGTTCCTGTCTAAAATCCCTTT
AATCGGCCTCCTGTTTAGCTC
CCGCTCTGATTCTAACGAGGAAAGCACGTTATACGTGCTCGTCAAAGCAACCATAGTAC
GCGCCCTGTAGCGGCGCATTA
AGCGCGGCGGGTGTGGTGGTTACGCGCAGCGTGACCGCTACACTTGCCAGCGCCCTAGC
GCCCGCTCCTTTCGCTTTCTT
CCCTTCCTTTCTCGCCACGTTCGCCGGCTTTCCCCGTCAAGCTCTAAATCGGGGGCTCC
CTTTAGGGTTCCGATTTAGTG
CTTTACGGCACCTCGACCCCAAAAAACTTGATTAGGGTGATGGTTCACGTAGTGGGCCA
TCGCCCTGATAGACGGTTTTT
CGCCCTTTGACGTTGGAGTCCACGTTCTTTAATAGTGGACTCTTGTTCCAAACTGGAAC
AACACTCAACCCTATCTCGGT
CTATTCTTTTGATTTATAAGGGATTTTGCCGATTTCGGCCTATTGGTTAAAAAATGAGC
TGATTTAACAAAAATTTAACG
CGAATTTTAACAAAATATTAACGTCTACAATTTAAATATTTGCTTATACAATCTTCCTG
TTTTTGGGGCTTTTCTGATTA
TCAACCGGGGTACATATGATTGACATGCTAGTTTTACGATTACCGTTCATCGATTCTCT
TGTTTGCTCCAGACTCTCAGG
CAATGACCTGATAGCCTTTGTAGAGACCTCTCAAAAATAGCTACCCTCTCCGGCATGAA
TTTATCAGCTAGAACGGTTGA
ATATCATATTGATGGTGATTTGACTGTCTCCGGCCTTTCTCACCCGTTTGAATCTTTAC
CTACACATTACTCAGGCATTG
CATTTAAAATATATGAGGGTTCTAAAAATTTTTATCCTTGCGTTGAAATAAAGGCTTCT
CCCGCAAAAGTATTACAGGGT
CATAATGTTTTTGGTACAACCGATTTAGCTTTATGCTCTGAGGCTTTATTGCTTAATTT
TGCTAATTCTTTGCCTTGCCT
GTATGATTTATTGGATGTTGGAATCGCCTGATGCGGTATTTTCTCCTTACGCATCTGTG
CGGTATTTCACACCGCATATG
GTGCACTCTCAGTACAATCTGCTCTGATGCCGCATAGTTAAGCCAGCCCCGACACCCGC
CAACACCCGCTGACGCGCCCT
GACGGGCTTGTCTGCTCCCGGCATCCGCTTACAGACAAGCTGTGACCGTCTCCGGGAGC
TGCATGTGTCAGAGGTTTTCA
CCGTCATCACCGAAACGCGCGAGACGAAAGGGCCTCGTGATACGCCTATTTTTATAGGT
TAATGTCATGATAATAATGGT
TTCTTAGACGTCAGGTGGCACTTTTCGGGGAAATGTGCGCGGAACCCCTATTTGTTTAT
TTTTCTAAATACATTCAAATA
TGTATCCGCTCATGAGACAATAACCCTGATAAATGCTTCAATAATATTGAAAAAGGAAG
AGTATGAGTATTCAACATTTC
CGTGTCGCCCTTATTCCCTTTTTTGCGGCATTTTGCCTTCCTGTTTTTGCTCACCCAGA
AACGCTGGTGAAAGTAAAAGA
TGCTGAAGATCAGTTGGGTGCACGAGTGGGTTACATCGAACTGGATCTCAACAGCGGTA
AGATCCTTGAGAGTTTTCGCC
CCGAAGAACGTTTTCCAATGATGAGCACTTTTAAAGTTCTGCTATGTGGCGCGGTATTA
TCCCGTATTGACGCCGGGCAA
GAGCAACTCGGTCGCCGCATACACTATTCTCAGAATGACTTGGTTGAGTACTCACCAGT
CACAGAAAAGCATCTTACGGA

TGGCATGACAGTAAGAGAATTATGCAGTGCTGCCATAACCATGAGTGATAACACTGCGG
CCAACTTACTTCTGACAACGA
TCGGAGGACCGAAGGAGCTAACCGCTTTTTTGCACAACATGGGGGATCATGTAACTCGC
CTTGATCGTTGGGAACCGGAG
CTGAATGAAGCCATACCAAACGACGAGCGTGACACCACGATGCCTGTAGCAATGGCAAC
AACGTTGCGCAAACTATTAAC
TGGCGAACTACTTACTCTAGCTTCCCGGCAACAATTAATAGACTGGATGGAGGCGGATA
AAGTTGCAGGACCACTTCTGC
GCTCGGCCCTTCCGGCTGGCTGGTTTATTGCTGATAAATCTGGAGCCGGTGAGCGTGGG
TCTCGCGGTATCATTGCAGCA
CTGGGGCCAGATGGTAAGCCCTCCCGTATCGTAGTTATCTACACGACGGGGAGTCAGGC
AACTATGGATGAACGAAATAG
ACAGATCGCTGAGATAGGTGCCTCACTGATTAAGCATTGGTAACTGTCAGACCAAGTTT
ACTCATATATACTTTAGATTG
ATTTAAAACTTCATTTTTAATTTAAAAGGATCTAGGTGAAGATCCTTTTTGATAATCTC
ATGACCAAAATCCCTTAACGT
GAGTTTTCGTTCCACTGAGCGTCAGACCCCGTAGAAAAGATCAAAGGATCTTCTTGAGA
TCCTTTTTTTCTGCGCGTAAT
CTGCTGCTTGCAAACAAAAAAACCACCGCTACCAGCGGTGGTTTGTTTGCCGGATCAAG
AGCTACCAACTCTTTTTCCGA
AGGTAACTGGCTTCAGCAGAGCGCAGATACCAAATACTGTCCTTCTAGTGTAGCCGTAG
TTAGGCCACCACTTCAAGAAC
TCTGTAGCACCGCCTACATACCTCGCTCTGCTAATCCTGTTACCAGTGGCTGCTGCCAG
TGGCGATAAGTCGTGTCTTAC
CGGGTTGGACTCAAGACGATAGTTACCGGATAAGGCGCAGCGGTCGGGCTGAACGGGGG
GTTCGTGCACACAGCCCAGCT
TGGAGCGAACGACCTACACCGAACTGAGATACCTACAGCGTGAGCTATGAGAAAGCGCC
ACGCTTCCCGAAGGGAGAAAG
GCGGACAGGTATCCGGTAAGCGGCAGGGTCGGAACAGGAGAGCGCACGAGGGAGCTTCC
AGGGGGAAACGCCTGGTATCT
TTATAGTCCTGTCGGGTTTCGCCACCTCTGACTTGAGCGTCGATTTTTGTGATGCTCGT
CAGGGGGGCGGAGCCTATGGA
AAAACGCCAGCAACGCGGCCTTTTTACGGTTCCTGGCCTTTTGCTGGCCTTTTGCTCAC
ATGTTCTTTCCTGCGTTATCC
CCTGATTCTGTGGATAACCGTATTACCGCCTTTGAGTGAGCTGATACCGCTCGCCGCAG
CCGAACGACCGAGCGCAGCGA
GTCAGTGAGCGAGGAAGCGGAAGAGCGCCCAATACGCAAACCGCCTCTCCCCGCGCGTT
GGCCGATTCATTAATG
