# Supplementary material for: Neuromelanin accumulation drives endogenous synucleinopathy in non-human primates
Source: Brain. 2023 Sep 28;146(12):5000–14. doi: 10.1093/brain/awad331 (PMC10689915; doi:10.1093/brain/awad331)
Supplement: awad331_Supplementary_Data [file awad331_supplementary_data.zip › brain-2023-00752-File011.pdf]

Supplementary Table 1: Animal records

Monkey	Gender	Weight & date of arrival	date of birth – date of sacrifice	Follow-up	Supplier
M307F8	Female	2.373 Kg (16/06/2020)	20/11/2017 – 02/03/2021	8 months	BioPrim (France)
M308F4	Female	2.430 Kg (16/06/2020)	21/11/2017 – 15/12/2020	4 months	BioPrim (France)
M309M8	Male	4.508 Kg (11/06/2020)	02/07/2017 – 02/03/2021	8 months	BioPrim (France)
M310M4	Male	3.783 Kg (11/06/2020)	07/07/2017 – 15/12/2020	4 months	BioPrim (France)
