# Supplementary figures and images for: Neuromelanin accumulation drives endogenous synucleinopathy in non-human primates
Source: Brain. 2023 Sep 28;146(12):5000–14. doi: 10.1093/brain/awad331 (PMC10689915; doi:10.1093/brain/awad331)

Supplementary Figure 1: Plasmid map for pAAV-CMV-hTyr

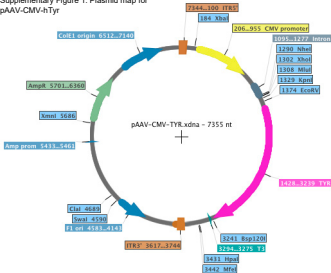

Supplement: awad331_Supplementary_Data [file awad331_supplementary_data.zip › brain-2023-00752-File009.pdf]
